# High-Temperature Mechanical Properties of NbTaHfTiZrV_0.5_ Refractory High-Entropy Alloys

**DOI:** 10.3390/e25081124

**Published:** 2023-07-26

**Authors:** Zhangquan Liu, Xiaohui Shi, Min Zhang, Junwei Qiao

**Affiliations:** Laboratory of High-Entropy Alloys, College of Materials Science and Engineering, Taiyuan University of Technology, Taiyuan 030024, China; liuzhangquan0020@link.tyut.edu.cn (Z.L.); sxhtough@126.com (X.S.); zm-821202@163.com (M.Z.)

**Keywords:** refractory high-entropy alloy (RHEA), mechanical properties, high-temperature strength, constitutive equation

## Abstract

The NbTaHfTiZrV_0.5_ is a refractory multi-principal-element alloy with high strength and good ductility at room temperature. It is important for possible high-temperature applications to investigate the deformation mechanism of the NbTaHfTiZrV_0.5_ alloy at different temperatures using tensile tests. In this investigation, the tensile tests were conducted at room temperature to 1273 K on sheet materials fabricated by cold rolling combined with annealing treatments. At 473 K, the NbTaHfTiZrV_0.5_ alloy exhibited a high tensile ductility (12%). At a testing temperature range of 673~873 K, the ductility was reduced, but the yield strength remained above 800 MPa, which is rare in most other alloys. The TEM investigations revealed that a dislocation slip controlled the plastic deformation, and the degree of deformation was closely related to the dislocation density. The true stress–strain curves of the alloy under different deformation conditions were obtained by tensile deformation at different deformation temperatures (673~873 K) and strain rates (0.001~0.0005 s^−1^). Experimental results were utilized to construct the parameters of a constitutive model based on a traditional mathematical model to predict the flow behavior at high temperatures. The excellent high-temperature mechanical properties of the NbTaHfTiZrV_0.5_ alloy will enable it to be used in several engineering applications.

## 1. Introduction

Demands for increased energy efficiency and pollution reduction have fueled research into new materials, particularly those utilized in the construction of gas-turbine engines, which have specialized features such as high temperature and corrosion resistance [1,2,3]. Nickel-based superalloys are mostly utilized for hot sections and can withstand temperatures of up to 1000 °C. However, the low melting points of pure nickel (1455 °C) and nickel-based superalloys (1250–1300 °C) restrict their usage at the higher operating temperatures required for more efficient propulsion performance [1,4]. As a result, designing novel elevated-temperature materials that can work at higher temperatures is crucial.

Compared with traditional refractory alloys or nickel-based alloys, a novel design strategy that involves mixing multicomponents, based on the concept of a high configurational entropy favoring the formation of a solid–solution phase, has recently attracted significant attention, and the resulting alloys are known as high-entropy alloys (HEAs) [5]. As a new class of metallic materials, HEAs or complicated concentrated alloys made up of four or more primary elements greatly increase the alloy design space [6,7,8]. HEA microstructures can be tailored to suit desired qualities, including single-phase solid solution HEAs [9,10,11] and HEAs with hierarchical microstructures [12,13,14,15]. Moreover, over a wide temperature range, an increasing number of appealing features in HEAs have been revealed, including an outstanding strength–ductility synergy at ambient temperature [16,17], extraordinary mechanical capabilities at cryogenic temperatures [18], and ultrahigh strength at extreme temperatures [19].

Refractory HEAs (RHEAs) stand out among various types of HEAs due to their unparalleled mechanical properties at high temperatures exceeding 800 °C, and even up to 1500 °C in some cases [20,21], making them viable options for high-temperature structural applications, outperforming typical Ni-based superalloys. Refractory high-entropy alloys, thanks to their high strength at high temperatures, high melting point, and high hardness brought by their resistance to friction and wear properties, may be used in aerospace engines, gas turbines, and the nuclear irradiation field. However, the reported literature has primarily centered on compression deformation investigations. For most RHEAs, due to their room-temperature brittleness, their tensile ductility is commonly negligible [22]. Therefore, improving the room-temperature plasticity of RHEAs is the premise of its high-temperature application.

The NbTaHfTiZr HEA has received a lot of interests among all RHEAs, since it has a totally disordered BCC solid-solution structure with a melting point of about 1800 °C [23,24]. At normal temperature and cryogenic temperatures, the NbTaHfTiZr RHEA combines excellent strength and ductility [25]. The NbTaHfTiZrV_0.5_ RHEA based on this alloy not only maintains excellent room-temperature plasticity but also further improves its strength and has a good cold workability [26]. In order to explore the potential of the NbTaHfTiZrV_0.5_ RHEA for high-temperature applications, it is very necessary to study its mechanical properties at high temperatures. Here, we explore the mechanical properties of the NbTaHfTiZrV_0.5_ alloy from RT to 1227 K and analyze its microscopic deformation mechanism. At the same time, we construct the constitutive equation of this alloy through a stress–strain curve in a deformation temperature range of 873–1173 K and a 0.001–0.1 s^−1^ strain-rate range and quantify the relationship between the strength and temperature.

## 2. Materials and Methods

The as-cast metal ingots were prepared by vacuum arc melting; all the used raw material elements had a high purity (>99.9 atomic percent, at%). Prior to arc-melting the raw materials, the residual oxygen in the arc-melter chamber was absorbed using a high-purity Ti-getter. The chemical composition of the metal ingots was NbTaHfTiZrV_0.5_. All ingots were flipped over and remelted more than 5–8 times to promote the homogeneity. The specific composition of the alloy is shown in Table 1. The weight of as-cast samples was 30 g. The ingots were then drop-cast into a copper mold that was water-cooled and had dimensions of 36 × 12 × 3 mm. Each as-cast sample was thermomechanically treated, which included cold rolling with a 50% reduction in thickness (RIT), stress relieving annealing (800 °C, 10 min), and water quenching.

For the uniaxial tensile tests, dog-bone-shaped specimens with gauge lengths of 10 mm and cross-sectional sizes of 31 mm were made using electro-discharge machining. The samples’ surfaces were polished with 180-, 400-, 800-, 1200-, 1500-, and 2000-grit SiC paper. Tensile tests were carried out on an Instron 5969 universal testing equipment. The tests were run at 298–1027 K at a strain rate ($\dot{\varepsilon }$) of 0.001–0.1/s. Infrared heating was used as the heating method, and after reaching the testing temperature, it was held there for five minutes before the tests. The surface of the test sample was sprayed with a boron nitride high-temperature-resistant paint to prevent oxidation. At least three samples were measured in each case to ensure the accuracy of the data. Nanoindentation experiments were performed in an instrument equipped (Bruker, Minneapolis, MN, USA) with a Hysitron TI Premier nanoindenter at room temperature. Indentation was performed in load-controlled mode with a maximum load of 5000 μN and a loading time of 2 s. To rule out any potential overlap of the deformed zone induced by the nearby indentations, tests were carried out at 10 μm intervals.

The crystal structures of as-cast ingots and the specimen after deformation were characterized by an X-ray diffractometer (XRD) using a Cu K-alpha radiation through a 2θ range between 20° and 120°, and the scanning rates were 9 2θ/min in all tests. The XRD equipment model was the Aeris (PANalytical B.V., Alemlo, The Netherlands). Scanning electron microscopy (SEM) and transmission electron microscopy (TEM) were used to analyze the microstructure of RHEA samples in diverse experimental scenarios. To be specific, we used a scanning electron microscope (SEM) equipped with a backscatter electron (BSE) mode to investigate the microstructures and structural evolution. The content of elements in each sample was further studied by energy dispersion spectroscopy (EDS). The SEM and EDS tests used the same equipment, the model was a PhenomWorld (Phenom, Eindhoven, The Netherlands).

For transmission electron microscopy characterization, the samples were mechanically reduced to less than 50 µm in thickness before being cut into 3 mm diameter discs for the TEM examination. Finally, the twinjet electropolishing was performed at 20 V and 25 °C using 5% perchlorate acid, 35% n-butanol, and 60% methanol. The model of the equipment used for the TEM was a JEM-2100.(JEOL, Tokyo, JAP).

## 3. Results

**Figure 1:**
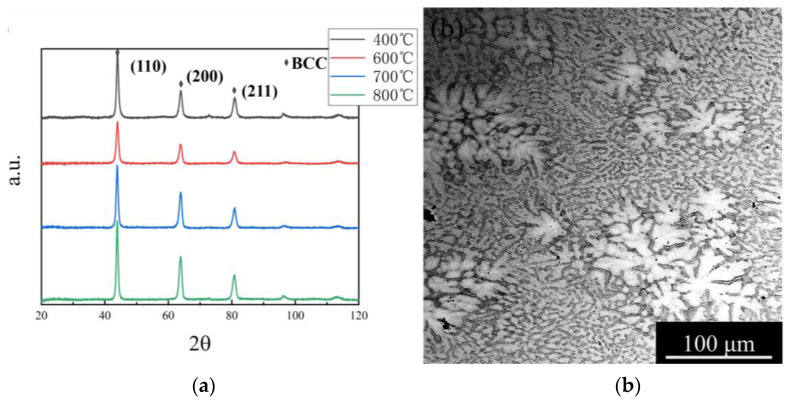
(**a**) XRD of NbTaTiZrHfV_0.5_ alloy at different temperatures. (**b**) SEM image of the NbTaTiZrHfV_0.5_ RHEA after cold rolling and heat treatment.

The XRD and microstructure analysis for the NbTaHfTiZrV_0.5_ HEA is presented in Figure 1. XRD tests were performed on all samples after high-temperature tensile tests and the results are shown in Figure 1a. It is obvious that all the samples were single-phase BCC structures, and the lattice constants were between 3.369 and 3.623 Å. Moreover, it can be preliminarily proved that there was no secondary phases or twins within the alloy after the high-temperature deformation. The lattice constants of the alloy after deformation at different temperatures are shown in the table below (Table 2). The lattice constants of the alloy were obtained by analyzing the XRD data with JADE 6.5 software. The error was no more than 5%. It can be found that with the increase in temperature, the lattice constant also increased slightly. As the temperature increased, the vibration of the atoms in the crystal increased, and the distance between the atoms increased, which led to the increase in the lattice constant. However, due to the short deformation time at high temperature, the change in lattice constant was not obvious. At the same time, the lattice constants of the components studied in this paper were compared with those of similar components, and it was found that the lattice constants of the components studied in this paper were not significantly different from those of these components. It also proved once again that the alloy studied was a single-phase BCC structure.

**Figure 2:**
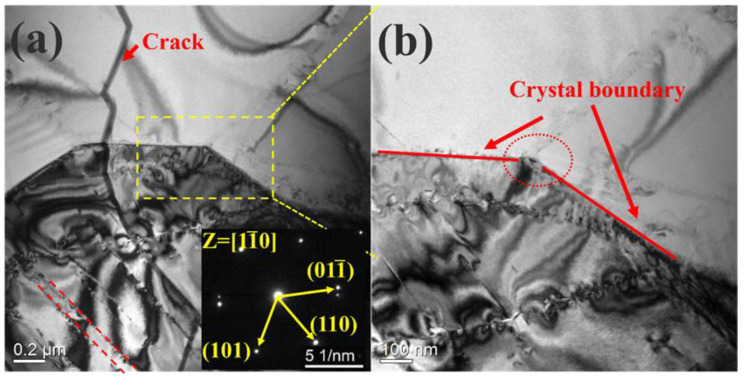
TEM images of the NbTaTiZrHfV_0.5_ RHEA after cold rolling and heat treatment. (**a**) cracks, grain boundaries and dislocation bands (red dashed lines) (**b**) Clearer triangular grain boundaries.

The SEM image of NbTaHfTiZrV_0.5_ HEA revealed a typical structure (Figure 1b). At temperatures between 400 and 800 °C, there was no significant difference in the microstructures of all tested samples. Similar to the as-cast situation, the alloy still had a typical dendritic structure [26]. The stress-relief annealing and high-temperature tensile tests did not change the microstructures of the alloy. In addition, the effect of a 50% cold rolling on dendrites could be found. The size of the dendrites was clearly not uniform, and the size of the largest was dozens of times that of the smallest. No significant cracks were observed on the SEM image, as opposed to the TEM image. In the transmission analysis, it was found not only dislocation bands but also a large number of grain boundaries. Figure 2b shows the obvious triangular grain boundaries. The discovery of grain boundaries indicates that although the alloy shows a dendritic structure on the SEM diagram, it is an equiaxial crystal under transmission cases. Therefore, it is considered that the dendritic structure of the alloy will not affect the mechanical properties of the alloy.

**Figure 3:**
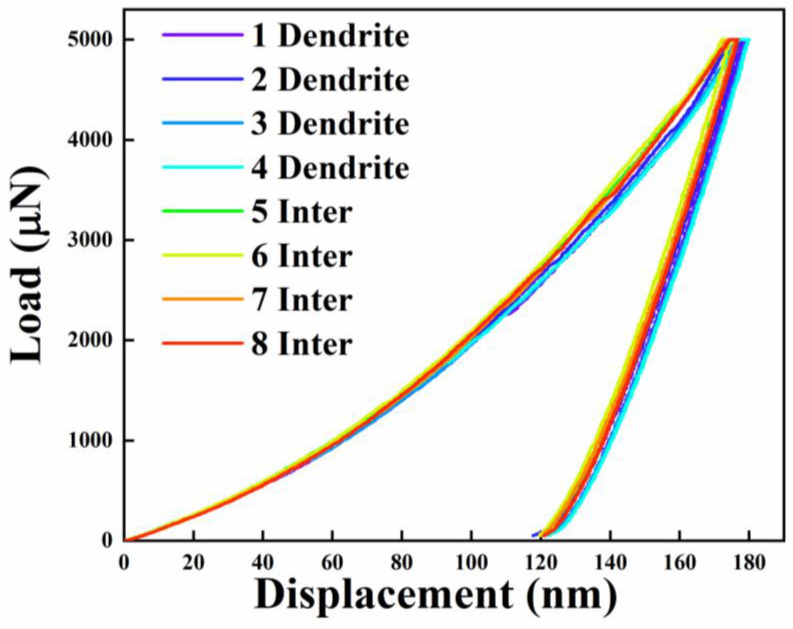
Nanoindentation load–displacement curves for the dendrite and interdendrite.

In order to test the micromechanical properties of the alloy, 16 tests were performed on the sample, with eight points on the dendrites and eight points between the dendrites. After the test was completed, we selected the best eight curves. Figure 3 shows the load–displacement curves of the dendrite and interdendrite nanoindentation. We can obviously see that whether it is a dendrite or interdendrite, the eight curves are highly overlapping, and it is almost impossible to distinguish the individual curves. The nanohardness of the alloy was 5.13 ± 0.01 GPa. This showed that the microhardness of the dendrites and interdendrites of the alloy were the same. From the results of the nanoindentation, it is reasonable to conclude that although the sample had a dendrite structure due to the element segregation, the micromechanical properties of the alloy were similar and did not need to be distinguished. The results of the nanoindentation and XRD were the same, proving that the alloy was unquestionably a single-phase structure.

**Figure 4:**
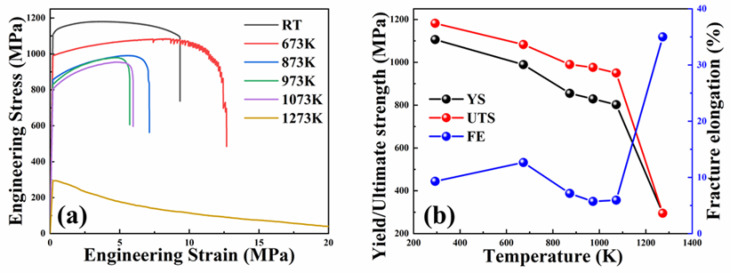
(**a**) Tensile stress–strain curves of the NbTaTiZrV_0.5_ RHEA at room temperature (RT) and high temperature (673–1273 K). (**b**) Temperature dependence of the yield strength (YS), the ultimate strength (UTS), and the fracture elongation (FE) for RHEA.

The tensile stress–strain curves of the NbTaTiZrV_0.5_ RHEA at room temperature (RT) and high temperature (673–1273 K) are presented in Figure 4a. The tensile properties of this HEA were shown to be in agreement with a previous study [26]. At room temperature, the cold-rolled alloy showed excellent tensile properties, with a yield strength approaching 1200 MPa and a plasticity reaching 8%. When the temperature reached 673 K, the strain–stress curve revealed a serrated flow behavior at a constant strain rate of 1 × 10^−3^ s^−1^, while the strength decreased, and the plasticity increased greatly. This occurred mainly in the softening phase, which may be related to the reason for the improvement in the plasticity [27]. When the temperature increased further, the yield strength and plasticity of the alloy decreased stably, but the decreasing range was small. The yield strength decreased significantly, and the plasticity rose to more than 30% when the temperature increased to 1223 K.

Figure 4b shows the temperature dependence of the yield strength (YS), the ultimate strength (UTS), and the fracture elongation (FE). When increasing the temperature, the yield strength gradually decreased, from 1200 MPa to less than 1000 MPa. It should be noted that a platform area was formed at 873–1073 K, and the strength changes could be ignored. According to the basic theory for a dislocation motion, the dislocation movement becomes easier at high temperatures [28,29]. That is to say, the strength of the alloy decreases as the temperature rises. The unusual “high-temperature plateau” identified in the NbTaTiZrV_0.5_ HEA, where the strength exhibited almost a temperature-independent behavior between 673 K and 1073 K, is of particular interest. It is unknown to date. In BCC alloys, similar results appear in only a few papers [30,31,32]. The yield strength of BCC pure metals and dilute BCC alloys is controlled by a screw dislocation motion through thermally triggered double-kink nucleation [21,33,34,35]. However, these theories cannot explain the mechanistic origin of the retained strength. Now, more recent studies speculate that the edge dislocations might have a thermal super-jogging, which makes them act as powerful barriers to the dislocation motion [21,36].

**Figure 5:**
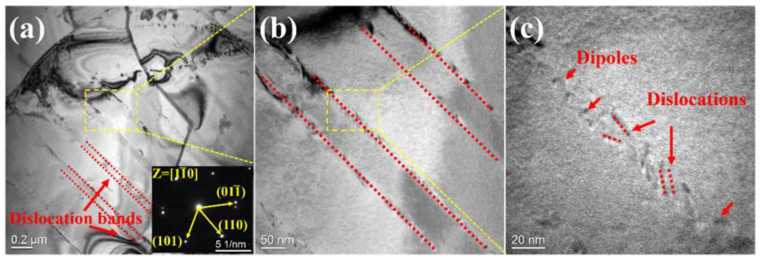
TEM images of the NbTaTiZrHfV_0.5_ RHEA and TEM image of the alloy before deformation. (**a**–**c**) show the morphology of dislocation bands at different observation scales (0.2 μm, 50 and 20 nm).

The differentials between the UTS and YS were about 100 MPa, and gradually widened as the temperature rose. Work hardening appeared in all curves from RT to 1073 K. We speculated that this was due to the fact that the heat treatment did not completely eliminate the residual stress of the alloy after cold rolling [37,38]. In order to prove this, the rolled samples were analyzed by transmission electron microscopy before a tensile test (see Figure 5). Some obvious dense dislocation bands were found in Figure 5a, and in addition, the diffraction pattern indicated that the alloy retained the structure of a single-phase BCC after the prior treatment. The region where the dislocation band was located was enlarged to further analyze its composition. In Figure 5b, it can be clearly found that the previously thought dislocation band was composed of two rows of dislocations. After a further enlargement, it was found that each dislocation band was composed of dense, short dislocations. This proved the previous conjecture that the residual dislocation after cold rolling caused a work hardening of the alloy.

**Figure 6:**
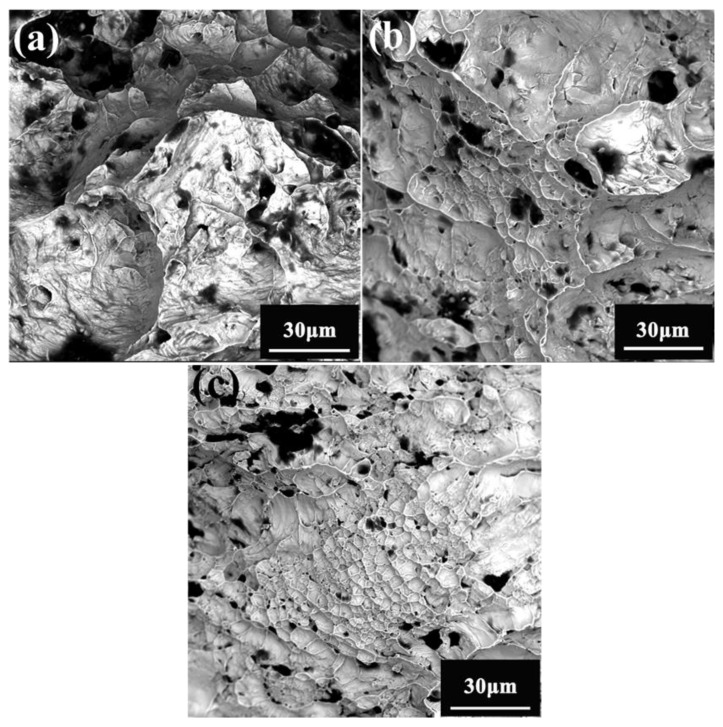
Tensile fracture morphology of the cold-rolled NbTaTiZrHfV_0.5_ HEA at different temperatures: (**a**) 693 K, (**b**) 873 K, and (**c**) 1073 K.

Compared with the strength, the plasticity change of the alloy was more complex, as presented in Figure 4b. Theoretically, with the increase in the temperature, the dislocation movement becomes easier due to the decrease in the resistance to the dislocation movement and the increase in the dislocation movement mode, and the elongation at the fracture of the single-phase alloys increases [39,40,41]. However, in this study, a low-plasticity plateau appeared when the temperature was between 873 K and 1073 K. In other words, in that temperature range, the alloy became more brittle compared to that at room temperature. Figure 6 shows the fracture diagram of the sample after tensile testing at 673, 873, and 1073 K. The specimens exhibited a ductile fracture at all three temperatures, and obvious dimples and fluvial patterns were observed from the fracture surface [42]. It is obvious from the figure that there is little difference between the fractures at all three temperatures. In addition, obvious black spots can be seen in all three images, which is the residue of the alloy oxidation. These oxides also show that the alloy was slightly oxidized during the tensile tests at high temperature.

**Figure 7:**
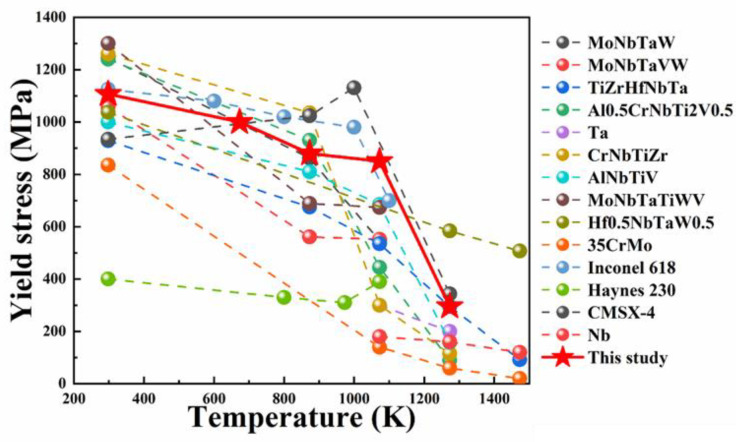
Tensile strength versus the testing temperatures of NbTaTiZrHfV_0.5_ with those of high-temperature metals and alloys, including conventional RHEAs [19,23,43,44,45,46,47,48], Ni-based superalloys [49,50,51,52], and refractory metals (Nb) [52].

In Figure 7, we compared the tensile strength of the NbTaHfTiZrV_0.5_ RHEA to that of other RHEAs [19,23,43,44,45,46,47,48], Ni-based superalloys [49,50,51,52], heat-resisting steels [53], and refractory metals [52] at extreme temperatures, demonstrating the NbTaHfTiZrV_0.5_ RHEA’s promise for high-temperature applications. The yield strength of the NbTaHfTiZrV_0.5_ alloy was higher in comparison to that of typical RHEAs. When compared to some of the most widely used Ni-based superalloys, the tensile strength of the NbTaHfTiZrV_0.5_ alloy was about 400 MPa higher than that of Haynes 230 and a little bit lower than Inconel 618 at the temperature range of RT-1073 K [Figure 5a]. Moreover, when the deformation temperature exceeded 1073 K, the strength of almost all metals and alloys dropped. This was due to the diffusion-controlled deformation mechanism activating above 0.5 to 0.6 *T_m_* and softening the materials as a result [54]. Therefore, the NbTaHfTiZrV_0.5_ RHEA not only had a high melting point but also had a high strength without any complicated treatment, which means that it has good prospects for high-temperature applications. 

**Figure 8:**
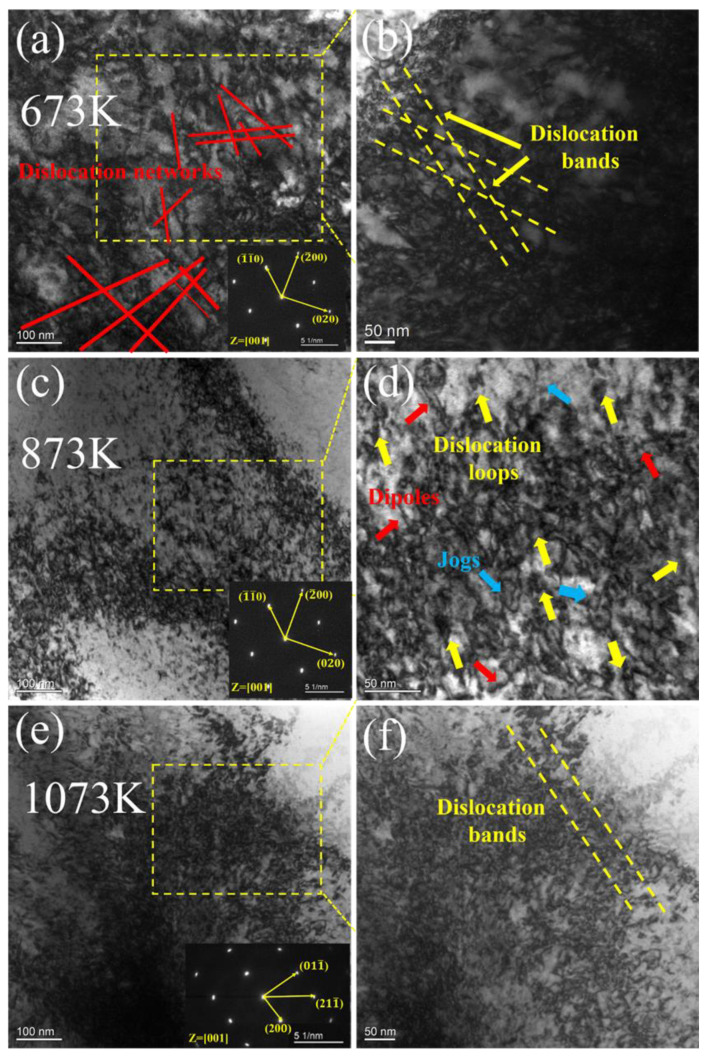
TEM bright-field image of the deformed sample of the Ti_37_V_15_Nb_22_Hf_23_W_3_ RHEA at different temperatures: (**a**) 673 K, (**b**) 873 K, (**c**) 1073 K. (**d**–**f**) are further enlarged images of (**a**), (**b**) and (**c**), respectively.

The microstructures of the NbTaHfTiZrV_0.5_ RHEA after experiencing tensile deformation at different temperatures until the final fracture were examined by using TEM (Figure 8). At all temperatures, the TEM images revealed only a large number of dislocations and no evidence of the appearance of deformation twins. Therefore, it indicated that the specimens’ deformation was only controlled by the dislocation slip. At 673 K, the dislocations were very dense, and many long and straight screw dislocations could be found, which were intertwined with each other to form a dislocation network, as shown in Figure 8a. Further magnified TEM images revealed more dense and complex dislocation structures. A large number of dislocation fragments and loops filled the entire image, making it difficult to distinguish individual dislocation structures. The high dislocation density and the existence of a dislocation network also confirmed the high plasticity of the alloy at 673 K. At higher temperatures (873 K), the dislocation density decreased, and the microstructure was observed to consist of dislocation bands and a large number of dislocation fragments (dipoles, jogs). At 1073 K, the microstructure was similar to that at 873 K except that the dislocation density was further reduced.

## 4. Discussion

In the previous results, we found that the crystal structure and microstructure of the alloy did not change significantly after rolling and heat treatment. After discussing the law of strength change with temperature and analyzing the results of the transmission electron microscopy, it is concluded that the deformation of the alloy at high temperature is mainly controlled by dislocation, and the morphology and density of the dislocation control the mechanical properties of the alloy. We have not discussed the plasticity change before, so the next focus is to analyze the change in alloy plasticity at high temperature and the reasons for that change. In high-temperature tensile tests, the strength of the alloy is more important, but the plasticity changes are equally important. In this study, it was obtained that the fracture plasticity of the alloy diminishes as the temperature rises (except at 673 K) (See Figure 4b). This trend is at odds with the experience with alloys in general, and this phenomenon may be the result of the following factors: First, the effect of high-temperature oxidation [55]. Despite the multiple treatments, the high-temperature oxidation during the stretching process cannot be avoided. When tested at high temperatures, oxygen-embrittling elements from the environment can rapidly permeate along grain boundaries, reducing the cohesiveness of grain boundaries and leading to the easy initiation of intergranular cracks. This dynamic oxygen embrittlement behavior has been reported in several other superalloys and intermetal complexes at a similar temperature range, which greatly limits their practical applications [55]. However, only a small number of impurities formed due to oxidation were found upon the fracture, which were not observed in either SEM or TEM observations. This indicates that the oxidative effect was not severe. Second, it may be due to a precipitation at high temperatures. The significant decrease in ductility at 1073 K is similar to that found in the TiZrHfNbTa alloy [56]. The precipitates from the grain boundary can damage the plasticity of the alloy. However, a previous analysis showed that the alloy had a single-phase BCC structure, and no precipitation was found by TEM. The possible reason is that the size of these precipitates was small, only a few nanometers in size, and it took a considerable time to find them in the field of view of TEM. This phenomenon will be the subject of another study.

**Figure 9:**
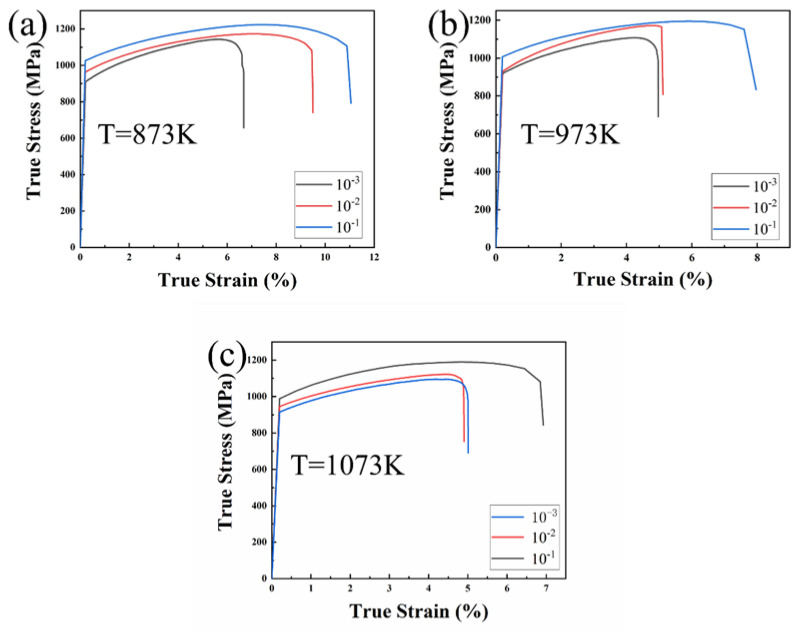
The tensile true stress–strain curves of the NbTaTiZrV_0.5_ RHEA at high temperature (873−1073 K) and different rates. (**a**) 873 K, (**b**) 973 K and (**c**) 1073 K.

Strength is one of the most important mechanical properties of materials used in high-temperature applications, and the ultimate tensile strength directly reflects the strength. The peak stress at different temperatures and strain rates can be predicted using the constitutive equation, because with an increasing temperature, the deformation mechanism changes significantly, which in turn increases the prediction error. To accurately predict the flow behavior, the prediction range was narrowed to 873 to 1073 K. Figure 9 shows the true stress–strain curves of the NbTaHfTiZrV_0.5_ RHEA at different deformation strain rates and temperatures. It was found that all curves had similar trends. That is, with the rise of the strain, the flow stress increased to the peak stress first and then decreased as the strain continued to increase. Before it reached the peak stress, the flow stress rose along with the strain. At this stage, the dislocation density increased due to deformation, the dislocation accumulation increased the deformation resistance, and a work hardening was the main rheological feature. When the flow stress achieved its maximal stress, it steadily declined as the strain increased. This is attributed to the dynamic recovery or dynamic recrystallization softening mechanism in the material exceeding the work-hardening mechanism, which is macrographically manifested as a gradual reduction in the flow stress [57]. In fact, the process of thermal deformation of materials is a competitive process of work-hardening and softening mechanisms, and the effects of hardening and softening reach an equilibrium when the flow stress reaches the peak stress [58]. The alloy’s peak stress drops with the increasing deformation temperature and increases with the increasing strain rate.

The constitutive relation is a model reflecting the relationship between the thermal deformation parameters and flow stress of deformed materials and is also the main basis for determining thermal deformation parameters. Sellars and Tegart proposed the Arrhenius equation, which includes the hot-deformation activation energy, strain rate, and deformation temperature. The specific equation is shown below [59]:

(1) $$\dot{\varepsilon }=A_{1}\sigma^{n_{1}}\exp\left(-\frac{Q}{RT}\right)$$

(2) $$\dot{\varepsilon }=A_{2}\exp\left(\beta \sigma \right)\exp\left(-\frac{Q}{RT}\right)$$

(3) $$\dot{\varepsilon }=A{\left[sinh\left(\alpha \sigma \right)\right]}^{n}\exp\left(-\frac{Q}{RT}\right)$$

$A_{1}$, $A_{2}$, A, α, β, n, and $n_{1}$ are material constants, *R* stands for the gas constant (8.314 J/mol^−1^K^−1^), *T* is the deformation temperature (K), *σ* is the peak flow stress (MPa), *Q* is the deformation activation energy (kJ/mol), and $\dot{\varepsilon }$ is the strain rate. In addition, the material constants  α, β, and ${\;n}_{1}$ have the following relationship:

(4) $$\alpha =\frac{\beta }{n_{1}}$$

The three equations of the Arrhenius equation have different application ranges. The power constitutive equation is applicable to low-stress conditions (ασ < 0.8), the exponential constitutive equation is suitable for high-stress conditions (ασ > 1.2), and the hyperbolic sinusoidal constitutive equation is a hybrid expression of the first two equations and is suitable for a wider range of stresses. Therefore, the hyperbolic sine equation was used to construct the constitutive model in this study. At the same time, the Zener–Hollomon parameter was introduced to measure the influence of the deformation temperature and strain rate on the flow stress during high-temperature deformation. The formula of the *Z* parameter is as follows [60]:

(5) $$Z=\dot{\varepsilon }\exp\left(\frac{Q}{RT}\right)$$

In order to facilitate the subsequent calculation of the constitutive equation parameters, the natural logarithm transformation of Equations (1)–(3) can be obtained as follows:

(6) $$\ln\dot{\varepsilon }=n_{1}\ln\sigma +\ln A_{1}-\frac{Q}{RT}$$

(7) $$\ln\dot{\varepsilon }=\beta \sigma +\ln A_{2}-\frac{Q}{RT}$$

(8) $$\ln\dot{\varepsilon }=n\ln\left[sinh\left(\alpha \sigma \right)\right]+\ln A-\frac{Q}{RT}$$

**Figure 10:**
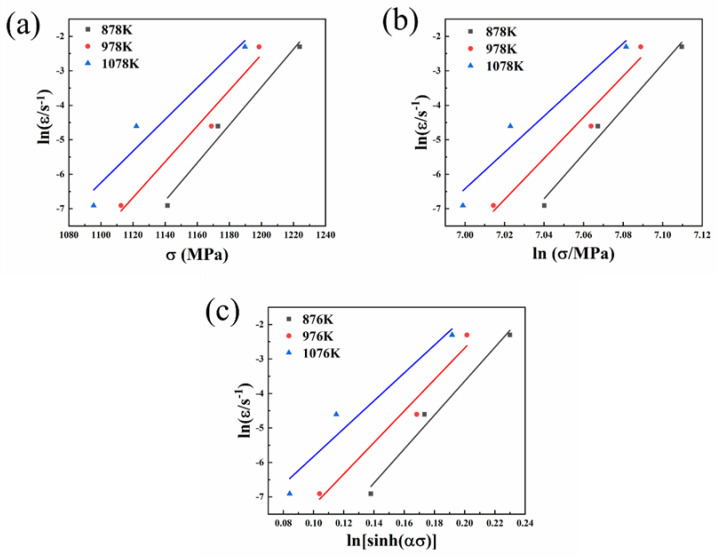
Relationship between peak stress and strain rate and temperature: (**a**) $\ln\dot{\varepsilon }-\sigma$ (**b**) $\ln\dot{\varepsilon }-\ln\sigma$ (**c**) $\ln\dot{\varepsilon }-\ln[sinh\left(\alpha \sigma \right)]$.

According to Formulas (6) and (7), $n_{1}\;$ and β represent the slopes of $\ln\dot{\varepsilon }$ and lnσ at different temperatures, respectively. Moreover, the value of *α* can be obtained by Formula (5). Figure 10 shows the linear-fitting diagram required to solve the material parameters in the constitutive equation. $\mathrm{The}\;\mathrm{values}\;\mathrm{of}\;n_{1}$, β, α, and nwere 59.18399, 0.050923, 0.00062, and 19.728, respectively. The deformation activation energy is an important physical parameter representing the plastic deformation of materials, and the solution equation is shown as follows [61]:

(9) $$\;Q=R\left[\frac{\partial \ln\dot{\varepsilon }}{\partial \ln[sinh\left(\alpha \sigma \right)]}\right]\left[\frac{\partial \ln[sinh\left(\alpha \sigma \right)]}{\partial \left(1/T\right)}\right]$$

**Figure 11:**
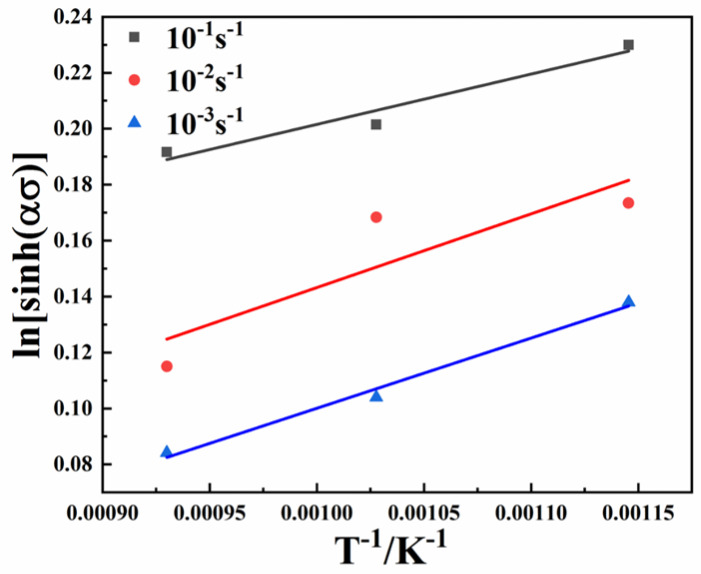
Relationship curves of $\ln[sinh\left(\alpha \sigma \right)]$ − *T*^−1^ of the NbTaTiZrV_0.5_ alloy at different strain rates.

The first and second terms of Formula (9) represent the linear-fitting slopes of $\ln\dot{\varepsilon }$ and $\ln[sinh\left(\alpha \sigma \right)]$ and $\ln[sinh\left(\alpha \sigma \right)]$ and $\left(1/T\right)$, respectively. The relationship curves of $\ln[sinh\left(\alpha \sigma \right)]$ − *T*^−1^ of the NbTaTiZrV_0.5_ alloy at different strain rates is presented in Figure 11. Taking the average of the slopes of the three fitting curves and substituting *n* and *R*, the deformation activation energy *Q* = 379 kJ/mol was obtained.

The solution of parameter *A* can be obtained by substituting Formula (5) into Formula (3) and taking the natural logarithm. The specific expression is as follows:

(10) $$\;Z\;=A{\left(\ln[sinh\left(\alpha \sigma \right)]\right)}^{n}$$

(11) $$\;\ln Z=\ln A+n\ln[sinh\left(\alpha \sigma \right)]$$

**Figure 12:**
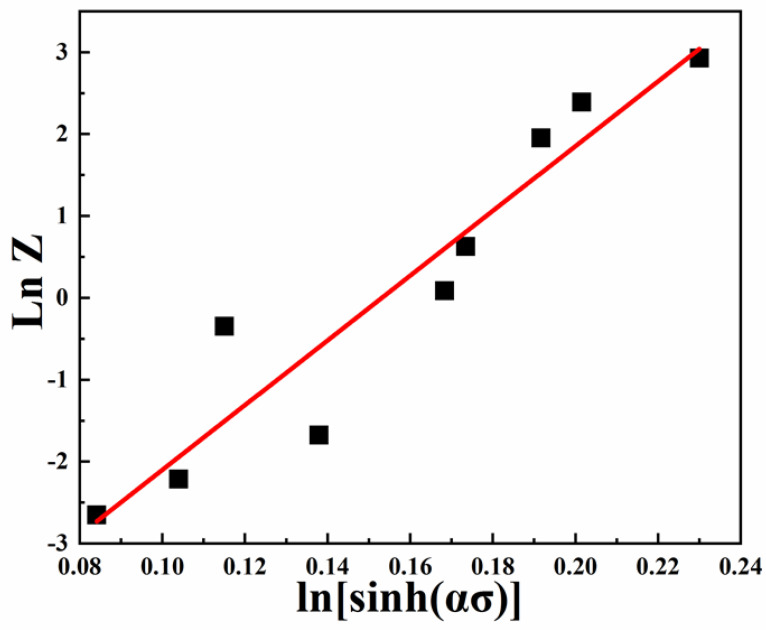
Relationship between ln*Z* − $\ln[sinh\left(\alpha \sigma \right)]$ of the NbTaTiZrV_0.5_ alloy at different strain rates.

Figure 12 plots the linear relationship between lnZ and $\ln[sinh\left(\alpha \sigma \right)]$, and the intercept represents the value of lnA, thus obtaining the value of *A* as e^6.0574^. Finally, the constitutive equation of alloy NbTaHfTiZrV_0.5_ is shown as follows:

(12) $$\sigma =\frac{1}{0.00062}\ln\left\{{\left(Z/e^{6.0574}\right)}^{1/19.7286}+{\left[{\left(Z/e^{6.0574}\right)}^{2/19.7286}+1\right]}^{1/2}\right\}$$

and $Z=\dot{\varepsilon }\exp\left(\frac{37,967}{RT}\right)$.

The following table (Table 3) shows the comparison of the experimental and predicted peak strength of the alloy at different temperatures and rates, and it can be found that the maximum error was about 100 MPa. This shows that the model obtained in this paper is reliable. The hyperbolic model has been used in other types of alloys, but it has been rarely used in refractory high-entropy alloys. This paper is perhaps the first use of this model, so it is relatively novel.

## 5. Conclusions

In this study, the mechanical properties of the NbTaHfTiZrV_0.5_ RHEA were tested at elevated temperatures. Additionally, XRD, SEM, and TEM were used to thoroughly characterize the samples both before and after testing. The results are summarized below:

(1) Over a wide temperature range of RT-1223 K, the studied alloy exhibited excellent tensile mechanical properties. From RT to 678 K, the strength of the alloy was maintained above 1000 MPa; At 878–1078 K, the strength variation was small and no less than 800 MPa, and it nearly exceeded most commercial alloys. Coupled with an excellent cold workability, the alloy has the potential to be used as a new generation of high-temperature materials for the preparation of high-temperature-resistant parts in aerospace.
(2) The true stress–strain curves of the alloy under different deformation conditions showed similar trends at temperatures between 873 K and 1073 K. That is, a work hardening occurred after yielding, and then a gradual softening until fracture. When the strain rate was constant, the yield strength and maximum strength of the alloy dropped as the temperature rose. When the deformation temperature remained constant, the strength grew in proportion to the strain rate.
(3) Strain-dependent constitutive equations based on hyperbolic sine laws were taken into consideration to forecast the flow behavior at various temperatures and strain rates. The stress values predicted by the constitutive equation were in good agreement with the experimental values.

## Figures and Tables

**Table 1:** The elemental composition of the NbTaHfTiZrV_0.5_ HEA samples.

Alloys	Concentrations (at.%)	Ti	Zr	Nb	Ta	V	Hf
TiZrHfNbTaV_0.5_	Cexp	18.09	17.54	18.33	19.13	8.95	17.99
	Cthe	18.18	18.18	18.18	18.18	9.09	18.18

**Table 2:** Lattice constants of NbTaHfTiZrV_0.5_ alloy after deformation at different temperatures and lattice constants of similar components.

Alloys	NbTaHfTiZrV_0.5_ (RT)	400 °C	600 °C	700 °C	800 °C
a (Å)	3.369	3.446	3.558	3.601	3.623
Alloys	NbTaHfTiZrMo_0.5_	NbTaHfTiZrMo_0.5_	NbTaTiVW	MoNbTaV	MoNbTaTiV
a (Å)	3.383	3.218	3.224	3.208	3.224

**Table 3:** The comparison of the experimental and predicted peak strength of the alloy at different temperatures and rates.

NbTaHfTiZrV_0.5_		873 K	973 K	1073 K
1 × 10^−3^ s^−1^	Exp	1141.55	1112.50	1095.49
	Cal	1019.31	994.84	975.68
1 × 10^−2^ s^−1^	Exp	1172.89	1168.89	1122.11
	Cal	1129.86	1096.62	1080.32
1 × 10^−1^ s^−1^	Exp	1223.76	1198.58	1189.75
	Cal	1247.42	1219.52	1195.81

## Data Availability

The data that support the findings of this study are available from the corresponding author upon reasonable request.
